# Prolonged Cadmium Exposure Alters Migration Dynamics and Increases Heterogeneity of Human Uterine Fibroid Cells—Insights from Time Lapse Analysis

**DOI:** 10.3390/biomedicines10040917

**Published:** 2022-04-16

**Authors:** Yitang Yan, Min Shi, Rick Fannin, Linda Yu, Jingli Liu, Lysandra Castro, Darlene Dixon

**Affiliations:** 1 Molecular Pathogenesis Group, Mechanistic Toxicology Branch, Division of the National Toxicology Program, National Institute of Environmental Health Sciences, National Institutes of Health, 111 TW Alexander Drive, Durham, NC 27709, USA; yitang.yan@nih.gov (Y.Y.); yu1@niehs.nih.gov (L.Y.); jingli.liu@nih.gov (J.L.); castro@niehs.nih.gov (L.C.); 2 Biostatistics & Computational Biology Branch, National Institute of Environmental Health Sciences, National Institutes of Health, 111 TW Alexander Drive, Durham, NC 27709, USA; shi2@niehs.nih.gov; 3 Molecular Genomics Core Laboratory, National Institute of Environmental Health Sciences, National Institutes of Health, 111 TW Alexander Drive, Durham, NC 27709, USA; fannin@niehs.nih.gov

**Keywords:** cadmium, fibroid, migration, time lapse, Ingenuity Pathway Analysis, straightness, heterogeneity, variance, carcinogenesis

## Abstract

Cadmium (Cd) is one of the most prevalent environmental heavy metal contaminants and is considered an endocrine disruptor and carcinogen. In women with uterine fibroids, there is a correlation between blood Cd levels and fibroid tumor size. In this study, fibroid cells were exposed to 10 µM CdCl_2_ for 6 months and a fast-growing Cd-Resistant Leiomyoma culture, termed CR-LM6, was recovered. To characterize the morphological and mechanodynamic features of uterine fibroid cells associated with prolonged Cd exposure, we conducted time lapse imaging using a Zeiss confocal microscope and analyzed data by Imaris and RStudio. Our experiments recorded more than 64,000 trackable nuclear surface objects, with each having multiple parameters such as nuclear size and shape, speed, location, orientation, track length, and track straightness. Quantitative analysis revealed that prolonged Cd exposure significantly altered cell migration behavior, such as increased track length and reduced track straightness. Cd exposure also significantly increased the heterogeneity in nuclear size. Additionally, Cd significantly increased the median and variance of instantaneous speed, indicating that Cd exposure results in higher speed and greater variation in motility. Profiling of mRNA by NanoString analysis and Ingenuity Pathway Analysis (IPA) strongly suggested that the direction of gene expression changes due to Cd exposure enhanced cell movement and invasion. The altered expression of extracellular matrix (ECM) genes such as collagens, matrix metallopeptidases (MMPs), secreted phosphoprotein 1 (*SPP1*), which are important for migration contact guidance, may be responsible for the greater heterogeneity. The significantly increased heterogeneity of nuclear size, speed, and altered migration patterns may be a prerequisite for fibroid cells to attain characteristics favorable for cancer progression, invasion, and metastasis.

## 1. Introduction

Uterine fibroids (leiomyomas) are common clonal smooth muscle neoplasms of the uterus that often appear during childbearing years. Uterine fibroids cause symptoms such as uterine bleeding and severe pelvic pain, resulting in infertility or major surgery [1]. As fibroid growth is hormonally regulated, environmental exposures to estrogen-like compounds, such as BPA, phthalate, and the metallohormone, cadmium, may contribute to the progression of fibroid tumors. It has been reported that blood Cd concentrations strongly correlate with fibroid volume [1]. Cd and its compounds were classified as a Group 1 human carcinogen by IARC and Cd accumulation is linked to cancer development [2]. In human populations, Cd exposure occurs primarily through dietary sources, cigarette smoking, and drinking water. Although Cd has no known physiological function, there is evidence to suggest that Cd is a potent metallohormone, mimicking the function of steroid hormones to promote the development of hormone dependent cancers [3]. The importance of estrogens in the etiology of breast cancer has shown that environmental exposures that mimic the effects of estrogens may pose a serious risk factor for disease development. Similar to estradiol, it has been shown that Cd can activate the classical ERα through the ligand binding domain or by membrane-associated ER, GPER1 [4,5,6]. Blood Cd levels have been reported to positively correlate with distant metastasis and clinical stage in breast cancers [7]. Exposure to Cd has also been reported to promote breast cancer cell migration and invasion in vitro [8]. However, the molecular mechanisms underlying Cd-induced migration and invasion of breast cancer cells have not been clearly understood [9]. 

It is estimated that Cd is efficiently retained in kidney with half-life about 10–30 years in human, owing partially to its low excretion levels [10]. Therefore, Cd exposure continues to be a major concern for public health. In vitro studies have shown that Cd exposure can malignantly transform normal human breast epithelial cells [11]. We have found that Cd can act as a metalloestrogen by nongenomic mechanisms upregulating GPER1 and EGFR, in addition to downstream events such as phosphorylation of Histone H3 resulting in proliferation of hormonally responsive human uterine leiomyoma cells [5,6]. We have also shown that fibroid cells subjected to Cd exposure for 2 months show enhanced migration potential, augmented anchorage-independent growth, and increased DNA synthesis [12], suggesting potential progression towards a cancer phenotype. 

Studies utilizing traditional imaging techniques have significantly contributed to our understanding of the interactions between cells and their extracellular environment. However, traditional techniques such as immunohistochemistry (IHC), immunofluorescence (IF), and electron microscopy (EM) typically require fixation, often involve sectioning, and eliminate the possibility to image live samples. Fortunately, current technology has pushed the envelope by adopting advanced modalities to image live samples over long periods and at higher spatial resolutions. The promise of these modalities has been illustrated by confocal microscopy to unravel migration dynamics that drive cancer invasion [13,14]. The dynamics of cell shape fluctuation, namely cellular morphodynamics, is of central importance for cellular function and development [15].

We hereby investigated the impact of prolonged Cd exposure on nuclear migration dynamics and morphodynamics. Our data showed that Cd exposure significantly enhanced nuclear motility and augmented nuclear size. Prolonged Cd exposure also significantly increased heterogeneity of nuclear instantaneous speeds and altered the migration behavior. The cell dynamics studies, along with mRNA profiling, strongly suggest that Cd exposure favors the progression of benign uterine fibroid tumor cells towards a cancer phenotype.

## 2. Materials and Methods

Cell cultures: A human uterine leiomyoma cell line immortalized via retroviral transfection of telomerase (ht-UtLM) was generated by our laboratory and maintained in culture as described previously [16]. All cultures were kept in a standard tissue culture incubator at 37 °C with 5% CO_2_.

Cd treatment: To derive 6-month Cd-exposed fibroid cells (CR-LM6), the ht-UtLM cells were maintained continuously in ht-UtLM standard culture medium [16] supplemented with 10 μM CdCl_2_ (Sigma-Aldrich, St. Louis, MO, USA, Cat# 439800) for 6 months. The culture medium was replaced with fresh medium every 3–4 days. The passage-matched control ht-UtLM cultures (ht-UtLM6) were generated by maintaining ht-UtLM in standard culture medium concurrently for 6 months.

Nuclear staining: Hoechst 33342 (2′-[4-ethoxyphenyl]-5-[4-methyl-1-piperazinyl]-2,5′-bi-1H-benzimidazole trihydrochloride trihydrate) (Thermo Scientific, Waltham, MA, USA, Cat#62249) was used to stain live cells cultured in glass-bottom dishes (MatTek Corporation, Ashland, MA, USA, Part No# P35G-1.5-14-C). Hoechst 33342 is a cell-permeable DNA stain that is excited by ultraviolet light and emits blue fluorescence at 460 to 490 nm. Before implementing time lapse imaging experiments, the cultured Cd-resistant fibroid culture exposed to Cd for 6 months (CR-LM6) and the passage-matched fibroid control culture for 6 months (ht-UtLM6) cells were stained with diluted Hoechst 33342 dye (1:10,000) in the standard cell culture medium for fibroid cells as previously described [16] for 2 h at 37 °C in an incubator.

Cell migration tracking: The CR-LM6 cells and passage-matched ht-UtLM6 control cells were grown in glass bottom dishes for 72 h to reach about 80% confluency and stained with Hoechst 33342. The time lapse images in 3 × 3 tile layout were recorded with a Zeiss LSM 880 confocal microscope (objective Zeiss Plan-Apochromate 20X/0.8 M27). Before starting the recording, the climate chamber in the confocal microscope was equilibrated to 37 °C and 5% CO_2_ for at least 30 min. For the time lapse observations, the time interval was set at 5 min and a total of 100 frames were recorded with Definite Focus turned on. The Definite Focus compensates for the sample drift and keeps sample in focus, especially for our long-term and multi-position time lapse experiments. The resulting time lapse files were stitched by Zen Black (Carl Zeiss Microscopy GmbH). The tracking was calculated with autoregressive motion algorithm with IMARIS 9.1.0 (Bitplane, Belfast, Northern Ireland). 

Data acquisition and statistical analysis: Time lapse images were analyzed with Imaris Surface model to derive parameter values including nuclear instantaneous speed, track straightness, track length, area, and shape. Due to the nonnormal distribution of the data, a Wilcoxon rank sum test was used to compare the two groups. The Fligner–Killeen test (in R package *stats*) was used for two-sample variance test. R version 4.02 was used in statistical analysis.

The conversion of nuclear area data from Imaris: The raw nuclear area values exported by Imaris are required to be divided by 2 to represent the correct nuclear size, because Imaris surface 3D model counts both bottom and top areas of a surface object.

Nuclear Track straightness: Nuclear Track Straightness (TrackStraightness) is defined as the ratio between Track Displacement and Track Length. The Track Displacement (TrackDisplacement) is the distance between the first and last nuclear object’s position. The Track Length (TrackLength) is the total length of displacements along the track (Imaris 9.1.0). 

Quantification of nuclear shape by Ratio C/B: The Length-C stands for Bounding Box Object Oriented (BoundingBoxOO) Length C, the length of the longest principal axis of a nucleus. The Length-B stands for BoundingBox OO Length B, the length of the second longest principal axis. The Ratio C/B is defined as Length-C/Length-B (Imaris 9.1.0).

RNA Isolation and NanoString Transcriptional profiling: Total RNA was extracted from three independent cell culture samples for the control and the Cd-exposed leiomyoma cells with Trizol™ Reagent and subsequently purified with RNeasy Mini kit. The NanoString Cancer Progression (NS_Cancerprog_C4972) and Cancer Pathways (NS_CancerPath_C2535) panels were used to determine the relative abundances of individual transcripts. DNA quality control, data normalization and Partek analysis, were performed as reported previously by our laboratory [12].

Ingenuity Pathway Analysis (IPA): The NanoString datasets containing gene identifiers and corresponding expression values were imported into IPA. A fold-change cutoff of 2.0 was adapted to identify molecules whose expression was significantly differentially expressed. Right-tailed Fisher’s exact test was used to calculate a *p*-value determining the probability that each biological function assigned to that network was due to chance alone [17]. 

## 3. Results

To understand the fundamental mechanisms of chronic Cd exposure-mediated carcinogenesis and malignant cellular transformation, we used benign uterine fibroid cells as a model to investigate alterations in cellular morphology (nuclear size and shape) and migration dynamics (instantaneous speed and track straightness) using time lapse imaging in a controlled environment. To increase cell population size, a 3 × 3 tile layout with 10% overlapping was adapted during the image acquisition stage. 

### 3.1. Cd Exposure Reduced Straightness of Migration Track

**Figure 1:**
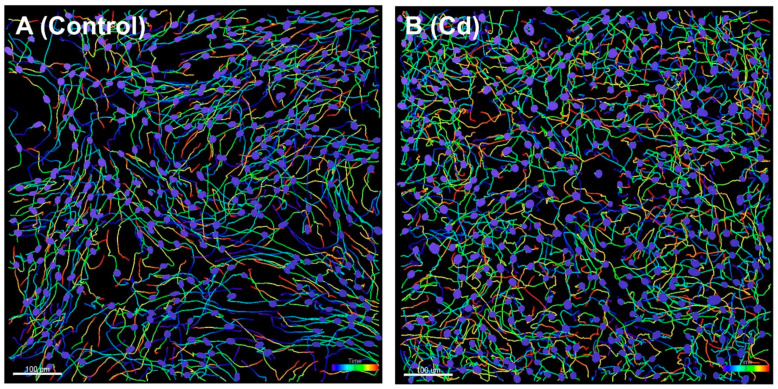
Full track display of nuclear surface objects to illustrate migration patterns. The images were captured at time frame number 50 (at 245 min). (**A**). Ht-UtLM6 tracks showed relatively parallel bundles in multiple regions. (**B**). CR-LM6 tracks showed relatively random and disorganized patterns. Scale bar = 100 μm.

Cell migration is a tightly regulated process. Cancer cells, in particular, exhibit a wide variety of migration mechanisms to direct random or directional persistent migration patterns [18]. To understand the fundamental mechanism of cancer progression, it would be ideal to develop a procedure to precisely quantify migration behavior. In this study, Imaris was used to analyze time series microscopic images to assess the effects of Cd exposure on migration straightness. When the two representative full track displays were used to detect nuclear surface objects in time lapse experiments, we were able to detect 478 and 530 tracks for the control and CR-LM6 cells, respectively. In the controls (Figure 1A), the ht-UtLM6 tracks showed parallel and coordinated track bundles in multiple sections of the image. However, the CR-LM6 tracks appeared to be more random, with obviously unparallel and less coordinated track bundles throughout the image (Figure 1B). Based on the track displays, the CR-LM6 cells appeared to migrate more haphazardly, and the prolonged Cd exposure may have reduced migration directionality due to the loss of cellular polarity [19,20].

**Figure 2:**
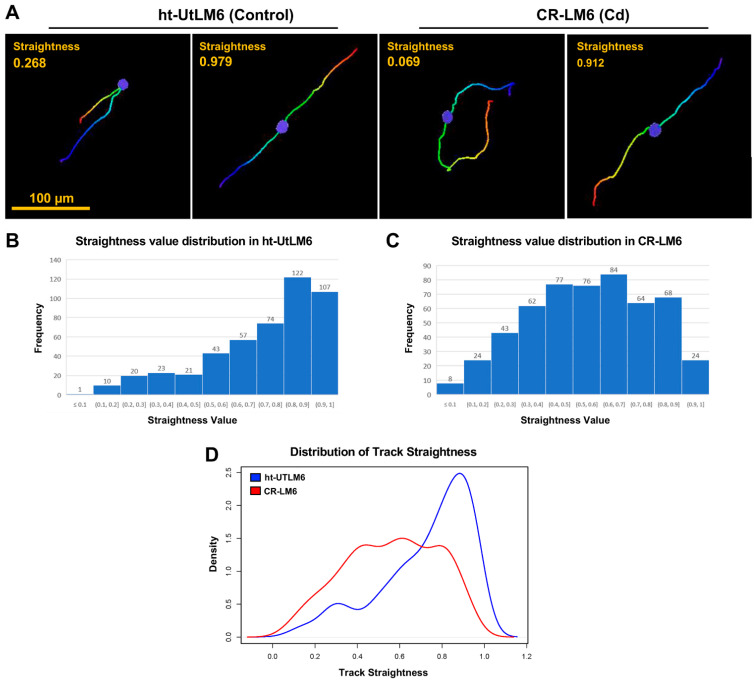
Distribution of Nuclear Track Straightness. (**A**) Tracks with various straightness values. These individual tracks were extracted from the full track displays in Figure 1. The Straightness is defined as the ratio between Track Displacement and Track Length. Scale bar = 100 μm. (**B**) Distribution of Track Straightness in ht-UtLM6. The data labels shown on top of the bars are the absolute counts. The Frequency in *Y*-axis denotes the number of counts within a specific bin. (**C**) Distribution of Track Straightness in CR-LM6. (**D**) Superimposed probability density function curves of Straightness values for both populations.

Nuclear Track Straightness is defined as the ratio between Track Displacement and Track Length. To introduce this concept, we presented two tracks with different Straightness values from each sample (Figure 2A). As indicated, the track was observed as a straight line if the associated Straightness value was greater than 0.9; however, the tracks appeared as a closed circle if the associated Straightness value was less than 0.1. Histograms were used to illustrate the distribution of the track straightness values of all the tracks for each sample. For the ht-UtLM6 sample, the distribution was significantly left-skewed with 22.3% of the total tracks (107/478) positioned in the bin with Straightness values of 0.9 or greater (Figure 2B). However, for the CR-LM6 population, there was no obvious skewness and only 4.5% of the total tracks (24/530) were positioned in the bin with Straightness values of 0.9 or greater (Figure 2C). The distribution can be represented by a probability density function, which shows the distribution of a continuous variable and the integral of the function between two points, i.e., area under the curve between two points, gives the probability that the variable falls within the interval. The difference in distributions can be easily visualized in the superimposed probability density curves (Figure 2D). Due to the skewed distribution of the data (Figure 2B,C), Wilcoxon rank sum test was used to compare the two samples. A significant lower Straightness value was observed in CR-LM6 population (mean 0.56; median 0.58) than ht-UtLM6 population (mean 0.72; median 0.79) with *p*-value 5 × 10^−33^ derived from the Wilcoxon rank sum test, indicating that the Cd exposure significantly altered the track straightness of fibroid cells. 

### 3.2. Cd Exposure Increased Nuclear Track Length

**Figure 3:**
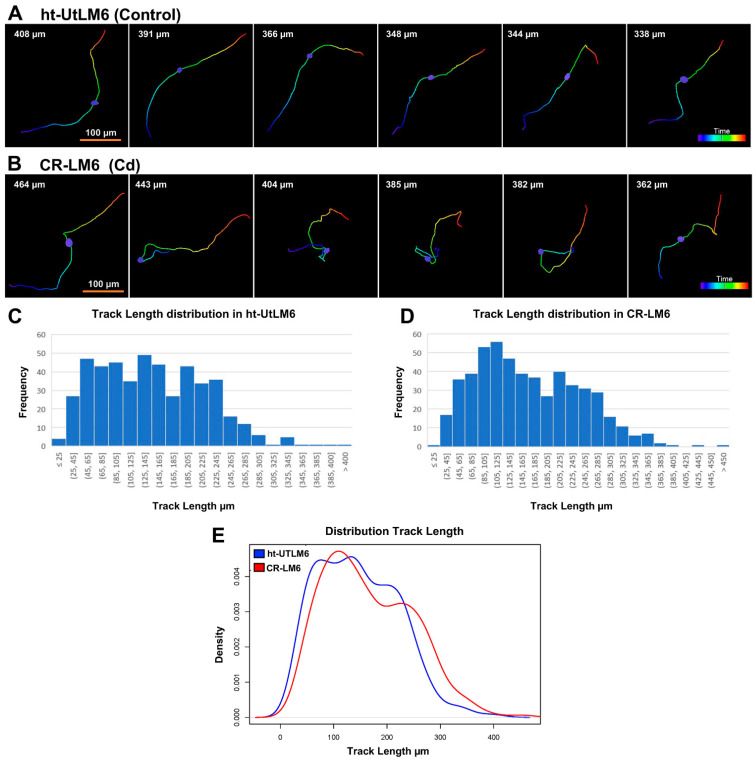
Track Length analysis. (**A**) The full track displays for the top 6 longest tracks. The Track Length values calculated by Imaris are displayed at the top-left. A. ht-UtLM6 tracks were relatively smooth and straight. (**B**) CR-LM6 tracks had sharp turns and sometimes loops. Scale bar = 100 μm. (**C**,**D**). The histograms of Track Length distribution for ht-UtLM6 and CR-LM6. (**E**) The probability density function curves of Track Length for the two populations.

The full track displays with the top 6 longest migration distances for both ht-UtLM6 and CR-LM6 populations are shown (Figure 3). It appeared that the track patterns were drastically different between the two cell populations. The ht-UtLM6 tracks appeared to be generally straighter than that of CR-LM6 (Figure 3A). Along CR-LM6 tracks, there were multiple occurrences of sharp turns and sometimes small loops (Figure 3B). As indicated by the Track Length values, the migration distances in CR-LM6 were consistently greater than ht-UtLM6 for the top 6 runners (Figure 3A,B). In ht-UtLM6 population, there was a total of 478 tracks with Track Length mean 146.2 µm and Track Length Standard Deviation (SD) 75.0 µm (Figure 3C). Whereas in the CR-LM6 population, there was a total of 530 tracks with Track Length mean 166.2 µm and Track Length SD 82.9 µm (Figure 3D). The maximum Track Length values in ht-UtLM6 and CR-LM6 were 408.3 and 464.3 µm, respectively. The two Track Length histograms and probability density curves were superimposed for direct comparison (Figure 3E). The Wilcoxon sum rank test comparing Track Lengths of the two populations gave a *p*-value of 2.9 × 10^−4^, indicating that the Track Length for CR-LM6 was significantly greater than the control. Comparison between Figure 2D and Figure 3E indicated that Track Straightness is a more sensitive end point to evaluate the effects of long-term Cd exposure on motility than the Track Length, which is also evidenced by the more significant *p*-value for the Straightness test.

### 3.3. Cd Exposure Increased Instantaneous Speed and Speed Variance

**Figure 4:**
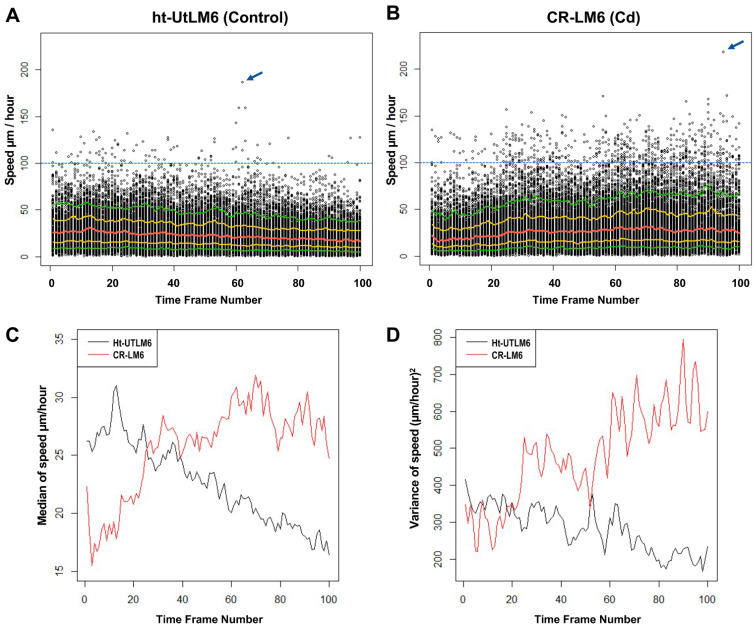
Prolonged Cd exposure enhanced instantaneous speed and increased speed variance. The instantaneous speed values were plotted against time frame numbers 0, 20, 40, 60, 80 and 100, corresponding to imaging times (in minutes) of 0, 95, 195, 295, 395 and 495, respectively. The five colored curves correspond to median (red), 25% and 75% quantiles (gold) and 10% and 90% quantiles (green). The speed reads above the blue dashed lines are greater than 100 µm/hour. The highest speed reads are marked by blue arrows. (**A**) ht-UTLM6. (**B**) CR-LM6. (**C**) The medians of instantaneous speeds at each time point were plotted against frame numbers. (**D**) The variances of instantaneous speeds at each time point were plotted against frame numbers.

To display the trend of nuclear speed fluctuations over time, the instantaneous speed values of all the nuclear surface objects at any given time point were plotted against time frame numbers. The instantaneous speed value refers to the speed read of a given nuclear object between the adjacent time frames calculated by the Imaris Surface model. There was a total of 32,274 and 33,584 speed reads in ht-UtLM6 and CR-LM6 populations, respectively. The speed datasets were exported from the Imaris Surface Tracking analysis. For the ht-UtLM6 dataset, the maximum speed read was 186.5 µm/hour, whereas for CR-LM6, the maximum was 218.1 µm/hour, as indicated by the blue arrows (Figure 4A,B). There were 82 and 496 speed reads greater than 100 µm/hour in ht-UtLM6 and CR-LM6 datasets (above blue dashed line), respectively. There was a general trend that in the CR-LM6 cell population, the speed reads were greater as the frame number increased (Figure 4B). Additionally, in CR-LM6, the spacing between 90% and 10% quantiles were progressively wider as time progressed, clearly indicating greater variance among the population, especially at the later time points. However, this was not true for ht-UtLM6, with the quantile spacings becoming smaller over time. In CR-LM6 cells, the median curve (red) showed a gradual slightly upward trend, whereas the 90% quantile (top green curve) showed a significant upward trend. However, in ht-UtLM6, all 5 curves consistently showed a downward pattern (Figure 4A,B).

At each time point, there were on average approximately 300 nuclear surface objects for each sample. We calculated the medians and variances of all the speed reads at each time point and plotted them against time frame number. It demonstrated clearly that the speed of the ht-UtLM6 population deaccelerated over time, whereas the speed of the CR-LM6 population accelerated over time (Figure 4C). Taking the median speed of the first 2 h for each nucleus, we compared between CR-LM6 and ht-UtLM6 cells. In the first 2 h, the median of the median speed of CR-LM6 was 21.0 µm/hr, while the median of the median speed of ht-UtLM6 was 28.5 µm/hr with Wilcoxon *p* value of 2 × 10^−12^. After the first 2 h, CR-LM6 had higher speeds (the median of the median was 28.4 µm/hr) than ht-UtLM6 (the median of the median was 22 µm/hr) with Wilcoxon *p* value < 2 × 10^−16^. Interestingly, the speed variance for ht-UtLM6 was reduced, whereas the speed variance for CR-LM6 increased over time (Figure 4D). The variances of the two groups were similar initially (Fligner–Killeen test *p*-value 0.26 at time frame 1), but by the end of the recording, the variance of CR-LM6 was significantly higher than that of ht-UtLM6 (Fligner–Killeen test *p*-value 3 × 10^−6^ at time frame 100). The speed variance results clearly indicated that Cd exposure enhanced speed heterogeneity in fibroid cells, which may be essential for Cd-mediated carcinogenesis. 

### 3.4. Cd Exposure Increased Nuclear Size and Nuclear Size Heterogeneity

**Figure 5:**
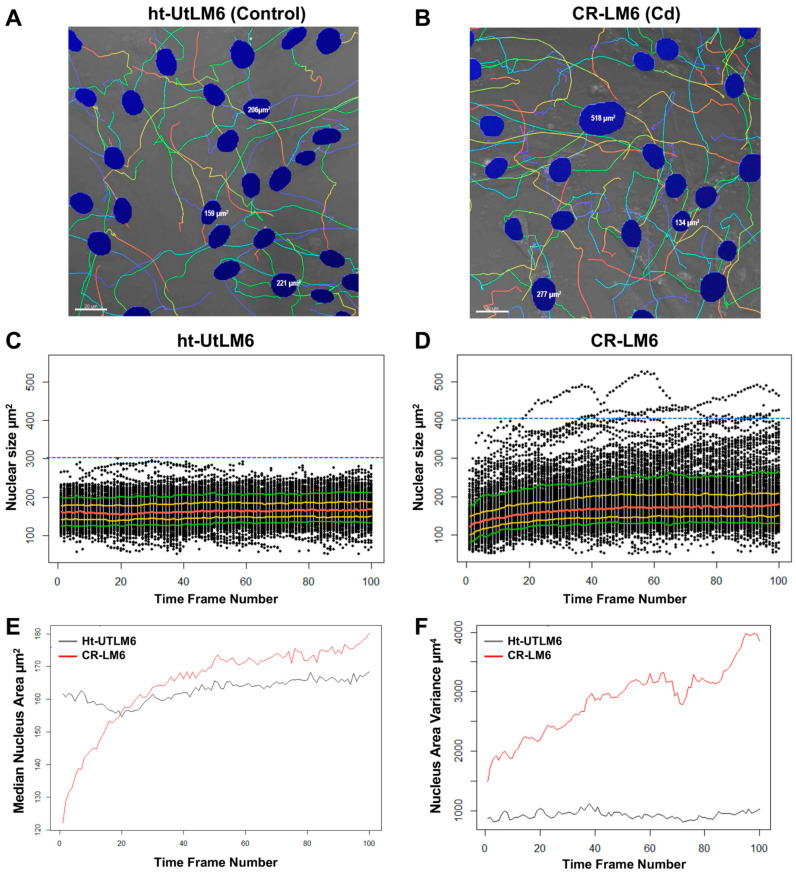
Cd exposure increased heterogeneity in nuclear size. The nuclear size, shape and migration tracks are shown. (**A**). ht-UTLM6. (**B**). CR-LM6. Scale bar = 20 μm. (**C**,**D**). Scatterplots to show the distribution of nuclear size over time. Note the presence of large nuclei on top of the plots. The five colored curves correspond with median (red), 25% and 75% quantiles (gold) and 10% and 90% quantiles (green). (**C**). ht-UtLM6. (**D**). CR-LM6. As shown by the blue dashed line, in ht-UtLM6 there was no nuclear objects with a size greater than 300 µm^2^, whereas in CR-LM6 there were 190 nuclear objects with a size greater than 400 µm^2^. (**E**). Medians of nuclear size at each time point were plotted against frame number. (**F**). Variances of nuclear size at each time point were plotted against frame number.

The CR-LM6 population displayed greater variation in nuclear size compared to control cells (Figure 5A,B). Specifically, we found greater nuclear heterogeneity in CR-LM6 with one nucleus measuring 518 µm^2^, which was significantly greater than adjacent nuclei (Figure 5B). To globally evaluate the fluctuations of nuclear size, we plotted all the nuclear size reads against time frame numbers.

In scatterplots (Figure 5C,D), the median values of nuclear sizes (red curve) in ht-UtLM6 were generally stable over time; however, the median values of CR-LM6 demonstrated an upward trend. As indicated by the greater gaps between the 10% and 90% quantiles (Figure 5D, green curves), the nuclear sizes in CR-LM6 showed much greater variation. In the CR-LM6 population, there were 1294 nuclear objects with sizes greater than 300 µm^2^ and 190 nuclear objects greater than 400 µm^2^ (Figure 5D). In contrast, the ht-UtLM6 population had no nuclear object with a size greater than 300 µm^2^ (Figure 5C). We calculated medians and variances of all the nuclear size values and plotted them against time frame numbers. The median of nuclear size of ht-UtLM6 population was relatively stable over time, fluctuating around 160 µm^2^. However, in CR-LM6, the nuclear size median increased significantly over time (Figure 5E), ranging from below 122 µm^2^ to above 180 µm^2^. In the first 95 min, the nuclear size of CR-LM6 (median 143.5 µm^2^) was smaller than that of ht-UtLM6 (median 155.5 µm^2^) and Wilcoxon *p*-value was 1.1 × 10^−6^. Afterwards, from 100 to 495 min, the nuclear size of CR-LM6 (median 168.8 µm^2^) was getting larger than ht-UtLM6 (median 162.1 µm^2^) with the *p*-value 3.6 × 10^−4^. Furthermore, the variance of nuclear size steadily increased in CR-LM6 from 1500 to 4000 µm^4^ (Figure 5F), suggesting that the stability of nuclear size in CR-LM6 cells was compromised following long term Cd exposure. Similar to our findings with the speed analysis, the cellular heterogeneity and temporal heterogeneity in nuclear size were also increased, which could contribute to the potential transition to a cancer phenotype induced by Cd exposure [21]. 

### 3.5. Cd Exposure Modified Nuclear Shape

**Figure 6:**
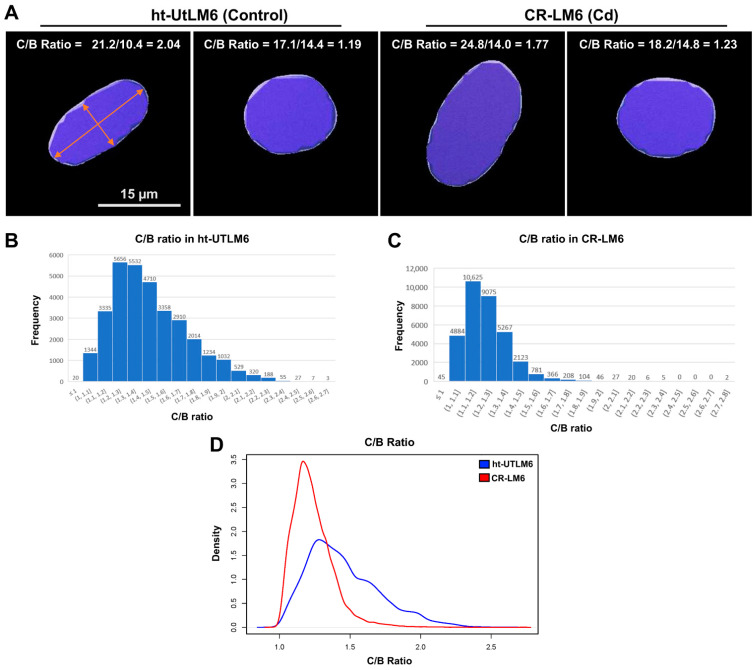
Cd exposure induced rounding of nuclei. (**A**) Shapes of selected nuclei with various C/B ratios from ht-UTLM6 and CR-LM6 populations. The C/B ratio is defined as ratio between the length of the longest principal axis of a nucleus and the length of the second longest principal axis. Scale bar = 15 μm. (**B**) The distribution of C/B ratio in ht-UTLM6. The data labels shown on top of the bars are the absolute counts. (**C**) The distribution of C/B ratio in CR-LM6. (**D**) The overlay of inset B and inset C by probability density function. The *x*-axis is the C/B ratio and *y*-axis is probability density.

The BoundingBox OO approach [22,23] was used to quantify the nuclear shape dynamics. The ratio C/B is a convenient measurement of nuclear shape. For a perfect mathematical circle, the C/B ratio equals to 1. The larger the C/B ratio, the more elongated the nucleus (Figure 6A). For both populations, there existed elongated nuclei (greater C/B ratios) and less elongated nuclei (lower C/B ratios) (Figure 6A).

As indicated in Figure 6B,C, the peak C/B ratio in the ht-UtLM6 population was located at the bin with C/B ratios of 1.2 to 1.3 with absolute counts 5656 (17.52% of the total nuclear objects), whereas CR-LM6′s peak was located at the bin with C/B ratios 1.1 to 1.2 with absolute counts 10,625 (31.63% of the total nuclear objects). The probability density distributions demonstrated that the C/B ratio in ht-UtLM6 had wider distribution than CR-LM6 (Figure 6D), indicating greater elongated nuclear shapes in the controls. The median C/B ratio in ht-UtLM6 was 1.40, which was significantly greater than the median (1.21) in CR-LM6 (*p* < 2 × 10^−16^). The Fligner–Killeen test of homogeneity of variances is also highly significant (*p* < 2 × 10^−16^) between ht-UtLM6 and CR-LM6. It appeared that Cd exposure promoted fibroid cells to form less typical elongated nuclei (with lower C/B ratios) than observed in typical smooth muscle cells histologically.

### 3.6. Cd Exposure Altered Orientation of Nucleus along Migration Track

**Figure 7:**
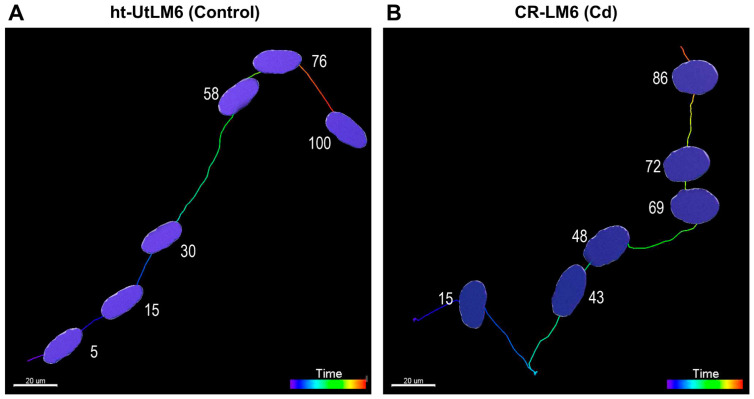
Cd exposure altered orientation of nuclei along migration tracks. The superimposed images show the location, shape, and orientation of nuclei. (**A**) The longest principal axis of ht-UTLM6 nucleus aligned with the direction of migration track at frames 5, 15, 30, 58, 76, and 100. (**B**) The longest principal axis of CR-LM6 nucleus was misaligned and occasionally perpendicular to the direction of migration track at frames 15, 69, 72, and 86. For nuclear orientations at all the time points, refer to supplementary videos (S1 and S2). Scale bar = 20 μm.

In the controls, the longest principal axis of the nucleus usually aligned well with the direction of migration track at all time points (Figure 7A; Supplementary Video S1, Track ID 32696), even at the location where the track was turning (time frame number 76). However, in the Cd-exposed fibroid cells, the longest principal nuclear axis did not always follow the direction of the track. For example, from frame 3 to frame 15, the longest nuclear axis gradually became perpendicular to the migration track. However, from frame 38 to 57, the longest axis aligned well with the track, but interestingly, from frame 69 to frame 91, the longest axis became perpendicular to the track again (Figure 7B; Supplementary Video S2, Track ID 33806). It appeared that the orientation of the longest axis of the nucleus was constantly changing in the CR-LM6 cells, which was not observed in the controls.

### 3.7. Gene Expression Profiling with NanoString PanCancer Panels

**Figure 8:**
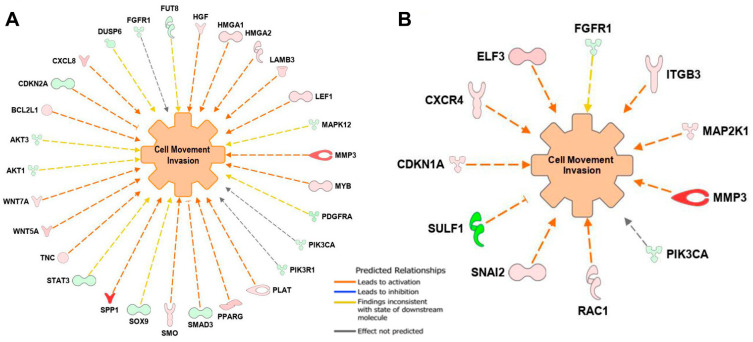
IPA predicted that Cd exposure stimulated migration and invasion in CRLM6 cells based on the directions of the gene expression changes. (**A**). IPA function analysis derived from the PanCancer Pathways panel dataset (*z*-score 2.30). (**B**). IPA functions analysis derived from the PanCancer Progression panel dataset (*z*-score 2.35). The dashed lines with an arrow indicate that upregulation of gene expression is predicted to promote cell movement. The dashed lines with a T-bar demonstrate that downregulation of gene expression is predicted to promote cell movement.

There are 580 and 600 probes for PanCancer Pathways and Progression Panels, respectively, with 132 probes overlapping. The data discussed in this publication have been deposited in NCBI’s Gene Expression Omnibus [24] and are accessible through GEO Series accession number GSE178790 (https://www.ncbi.nlm.nih.gov/geo/query/acc.cgi?acc=GSE178790, accessed on 29 October 2021). The datasets derived from PanCancer Pathways and PanCancer Progression Panel were analyzed by Ingenuity Pathway Analysis (IPA) for downstream functional analysis. For the Pathways Panel, the direction of gene expression changes was predicted to promote cellular movement, and specifically, the invasion of tumor cell lines (Functions Annotation) with activation *z*-score 2.30 and overlap *p*-value 1.63 × 10^−17^. In the network, 19 of 29 genes had measurement directions consistent with increase in tumor cell invasion (Figure 8A). For the Progression Panel, the direction of the gene expression favored cellular movement and, specifically, invasion based on data reported and curated for breast cancer cell lines (Functions Annotation) with activation *z*-score 2.35 and overlap *p*-value 1.7 × 10^−12^. In the network, 9 of 11 genes had measurement directions consistent with activation in the invasion of breast cancer cell lines (Figure 8B). An absolute *z*-score of ≥2 is considered statistically significant in IPA [17]. Therefore, based on the gene expression profiling of the two panels and IPA downstream effect analysis, the cell movement and invasion functions were significantly stimulated in Cd-exposed fibroid cells. 

Based on IPA analysis, the expression levels of *SPP1*, *MMP3*, and *SULF1* were significantly altered in CR-LM6 (Table 1). Specifically, the expression of both *SPP1* and *MMP3* was upregulated more than 15-fold with statistical significance, due to Cd exposure. Similar to ECM transcript data in our 2-month Cd exposure study [12], the expression of *MMP1*, *MMP3* and *MMP10* were all significantly upregulated in CR-LM6 (Table 2). Additionally, the expression of the collagen family members identified in our previous study [12] were also mostly inhibited, as shown in Table 2. However, the transcripts *COL1A1, COL5A1, COL4A1, COL11A1* were not differentially expressed in CR-LM6. The *COL7A1* was identified as a new downregulated transcript (reduced by more than 3-fold) in CR-LM6 (Table 2). Interestingly, *LAMA3* and *LUM*, two essential ECM components, were significantly downregulated in both 2-month and 6-month Cd-exposed fibroid cells [12].

## 4. Discussion

Time lapse microscopy has offered researchers vast amounts of live cell imaging data to explore. Historically speaking, in the early stage of migration studies, greater efforts were devoted to optimizing on track extraction using a plethora of segmentation and tracking algorithms. However, biologically relevant conclusions extracted from time-lapse imaging experiments, such as migration directionality or heterogeneity of migration patterns would be more meaningful. Additionally, there should be more of an effort to make data and codes openly available [25]. In this study, we focused our efforts on parameters beyond migration distances (track length), aiming to unravel the fundamental migration dynamics induced by prolonged Cd exposure in uterine fibroid cells.

We found that unexposed fibroid cells showed significant migration directionality; however, Cd exposure significantly reduced migration directionality as quantified by Track Straightness. Additionally, the average migration distance of the Cd-treated fibroid cell populations was significantly greater than that of untreated fibroid cells. It has been suggested that directional cell migration orients ECM fibrils to drive tissue shape changes during morphogenesis. Proper ECM expression levels and fibril alignment are required for maintaining migration and directionality in cell migration patterns [26]. In this study, we observed that Cd exposure dramatically reduced the expression of ECM components, the relative abundance of each ECM component, and thus impacting the ECM topography and biomechanical rigidity. For example, the gene expression levels of collagens, laminin, and lumican were grossly reduced ranging from about 2-4fold in our study. Additionally, Cd exposure significantly stimulated the expression of MMPs, specifically, *MMP1*, *MMP3* and *MMP10* by approximately 2-16fold, which may further contribute to the breakdown of ECM fibrils. The binding of ECM proteins to cell surface integrins and other receptors promotes a variety of cellular responses including adhesion, migration, survival, and proliferation [26]. It is well known that Rac activity promotes the formation of peripheral lamellae and membrane ruffles that promote random cell migration and may serve as a switch between patterns of cell migration [27]. Factors such as topography of ECM, cellular polarity, integrin trafficking, and actomyosin contraction converge on the regulation of the Rho family of GTPases [28]. Uterine fibroids feature excessive ECM, overexpression of collagens, increased stiffness, and altered mechanotransduction signaling. Fibroid growth can be stimulated by progestins. Cells cultured on stiff substrate such as polystyrene had enhanced PRB (progesterone receptor B) activation via a mechanism that required MEK 1/2 and AKAP13/RhoA/ROCK signaling. Estrogen and progesterone signaling, including Rho-associated coiled-coil kinase (ROCK), have a central role in regulating fibroid growth and ECM accumulation [29]. Prolonged Cd exposure may interfere with the above factors, especially the ECM topography and the loss of ECM contact guidance [12,30], converging on the Rho family of GTPases and boost migration randomness.

Speed analysis data showed that Cd exposure not only increased the instantaneous speed, but also enhanced the variation of the instantaneous speed over time, especially at later time points. The increased speed heterogeneity in CR-LM6 may be due to the lack of contact guidance conferred by extracellular matrix or the increased ECM anisotropy [30]. It is well established that cell migration is guided by anisotropic topographical features (i.e., structures with different geometric properties in different directions) of the substrate—a phenomenon which is defined as contact guidance. Moreover, cells dynamically adjust their migration mode to avoid cell bending due to substrate curvature on concave and convex surfaces [30]. Indeed, we demonstrated in this current and previous studies that chronic Cd exposure significantly reduces the expression of ECM components such as collagens, which are critical for contact guidance [12]. The collagens constitute a family of related proteins that are assembled into a variety of supramolecular structures in extracellular matrices. The collagen family comprises 28 members and play essential roles in regulating cell mechanical properties, shape, migration, and proliferation by interacting with multiple cell receptors [31]. The average nuclear size in fibroid cells became generally larger after Cd exposure. Additionally, with Imaris BoundingBox analysis, Cd exposure also rendered nuclei to become less elongated, which is typically the shape of a smooth muscle cell, to a more oval to rounded shape with the difference being statistically significant based on measurements of 60,000 plus nuclear objects. The larger nuclear size and increased nuclear morphological variation in CR-LM6 may be associated with greater chromosome instability and metastasis potential [32]. The interplay between chromatin’s compaction, spatial organization, and mechanics control nuclear size, shape, and function. An imbalance between lamin, chromatin and cytoskeleton has been reported to induce abnormal nuclear shape, which can disrupt chromatin functions, cause nuclear rupture, and increase DNA damage [32]. Heterochromatin mechanics may also influence the regulation of nuclear shape. Alterations in the amount of compact heterochromatin and loose euchromatin are commonly observed in cancers [33]. Therefore, due to the significant alteration of the nuclear shape and size, Cd exposure could likely interfere with homeostasis between lamin, chromatin, and cytoskeleton. In mussels, a marine invertebrate, it has been reported that industrial pollution causes Cd bioaccumulation in the testis. A positive correlation between Cd levels in testes and metallothionein expression was observed in mussels growing in contaminated waters with 1.5–10 µM CdCl2. Protamine-like proteins, the major basic nuclear components of mussel sperm chromatin, were found in an aggregated form with reduced DNA binding affinity in exposed mussels. These authors found that CdCl_2_ had a high affinity to DNA, interfered with sperm chromatin integrity, induced errors in DNA synthesis, and resulted in male infertility in the mussel population [34]. Additionally, CdCl_2_ has been shown to induce conformational changes in sperm protamine-like proteins and alter chromatin organization in mussels [35]. These findings indicate that Cd exposure can clearly modify chromatin structure and nuclear function. Alterations in nuclear size, shape, and function will have important implications for cancer progression. 

Cd exposure significantly promoted nuclear heterogeneity, as demonstrated by a Fligner–Killeen test of homogeneity of variances on nuclear size, shape, and speed in our datasets. Nuclear pleomorphism (variation in nuclear shape, size, and orientation) is an important constituent of cancer grading schemes. Computer-extracted features such as nuclear shape and orientation from hematoxylin- and eosin-stained tissue slides was used to help stratify patients into distinctive outcome groups. The histomorphometric features of nuclear shape and orientation are also strongly predictive of patient survival in breast cancer [36]. In many aggressive tumors, tissue structures become poorly differentiated as a result of rapid disorganized cell growth, promoting the formation of highly irregular nuclear patterns. Disorganized nuclear orientation and shape has been shown to be critical for progression in urothelial and breast cancer [36]. Intratumor heterogeneity implies that subpopulations of cancer cells that differ genetically coexist in a single tumor. Greater tumor heterogeneity drives cancer progression, metastasis, and treatment resistance [37].

In any cell population, heterogeneity is intrinsic in nature. Cancer cell populations typically display significant heterogeneity even when the cancer cells are derived from a single progenitor clone. Both cellular heterogeneity and temporal heterogeneity contribute to the overall heterogeneity of a cell population. The cellular heterogeneity refers to the time-independent cell-to-cell variation (population noise), while the temporal heterogeneity designates the temporal fluctuations of single cells (temporal noise) [21]. In our speed and shape analyses, both population heterogeneity and temporal heterogeneity components were identified in the time-series datasets. For example, in the nuclear size analysis, the temporal variance of the nuclear size increased steadily and sequentially. This temporal heterogeneity can derive from several factors such as various stages of cell cycle [38], circadian rhythm [39], and the levels of ATP [40] or Ca^2+^ [41]. A single cell can undergo temporal transitions between fast and slow migration states [42]. In order to quantify the migration behavior of lung cancer cells, both population and temporal heterogeneity were taken into account to elucidate the migration behavior [21]. Therefore, it is of critical importance to develop approaches beyond population averaging to address heterogeneity in cell migration [21] to understand the effects of toxicant exposures, such as Cd, on cancer cell progression and potential for increased motility and metastasis.

Our video recordings showed that the nuclear orientation in relation to the migration track changed dramatically in the Cd-exposed fibroid cells during cell migration in the time span of 8 h. It appears that Cd exposure not only interfered with nuclear speed and shape, but also effectively altered the orientation of the nucleus within a cell relative to migration direction. Interestingly, the cancerous prostate glands are usually less organized, resulting in more chaotic orientation in the adjacent nuclei. It was reported that the prostate cancer morphology could be quantitatively characterized with cell orientation entropy, a novel feature descriptor to describe cancer cells, aiming at modeling disorder of cell/nuclear orientation within local neighborhoods and predicting cancer recurrence [43]. In CR-LM6, nuclear orientation appeared to be disorganized in multiple locations along a migration track, mimicking the irregular nuclear organization patterns observed in aggressive cancers [19]. To maintain cell polarity, the centrosome is required to be positioned between the cell leading edge and nucleus [19]. This polarity is achieved by moving the nucleus backwards because of actin retrograde flow [19,20]. Thus, the directional cell migration is regulated by cytoskeleton-mediated nuclear relocation process within migrating cell. Cd exposure may have greatly interfered with the nuclear relocation process due to the significant reduction in migration directionality and disorientation of nuclei along the migration tracks. During normal cell migration, the position of the nucleus within a cell may have to be constantly monitored and adjusted by relocation processes to maintain directionality and orientation.

Our RNA profiling analysis showed that the expression directions of significantly altered genes in the dataset stimulated migration and invasion. SPP1 has cytokine activity and is capable of binding to the ECM via its integrin-binding sequence to promote cell migration. SPP1 also activates ROCK signaling via the FAK/PI3K/AKT pathway, thereby facilitating cancer invasion through lamellipodia formation [44]. In SCLC (small-cell lung cancer) patients, SPP1 is one of the most upregulated genes to stimulate epithelial–mesenchymal transition (EMT) [45]. SPP1 is also widely known to contribute to the migration of breast cancer cells to bone [46]. MMP3 is a zinc-dependent proteolytic enzyme capable of modifying extracellular matrix. MMP3 overexpression is associated with cancer metastasis, development of pre-malignant and malignant lesions, spontaneous neoplastic progression, and genomic instability. MMP3 has been linked to metastasis by promoting proteolysis of SPP1 into an active form. Human sulfatase 1 (SULF1) is capable of desulfation of heparan sulfate proteoglycans (HSPGs), which serve as co-receptors for growth factors and cytokines. In the majority of cancer cells, SULF1 is downregulated, and the suppressed SULF1 expression stimulates migration, enhances invasion and promotes proliferation [47]. Prostate cancer cells show metastatic tropism for bone marrow; over 80% of the patients who succumb to the disease possess bone metastases. A dynamic crosstalk between prostate cancer cells, cancer-associated fibroblasts (CAFs), and tumor-associated macrophages (TAMs) impacts cancer behavior. In cancer-stroma-macrophage triculture models, it was shown that the reduction in SULF1 stimulate the progression of bone metastasis in prostate cancer [48]. Following Cd exposure, the direction of the most differentially expressed genes such as *SPP1*, *MMP3* and *SULF1* in fibroid cells favored cellular migration, proliferation and cancer progression as predicted by IPA pathway analysis. 

In this study, we have demonstrated that additional features beyond cell painting [49], such as speed, track straightness and nuclear orientation, can be extracted from time lapse microscopic studies to dynamically evaluate the impact of chemical exposure. The increase in the ability of tumor cells to migrate, along with the increase in the heterogeneity of morphodynamic, are key characteristic properties of cancer and metastasis. The increased heterogeneity of nuclear size and cell speed, as well as the disorientation of nuclei, may be a prerequisite for Cd-exposed cells to navigate their microenvironment more efficiently and progress towards a cancer state. Our RNA profiling analysis suggest that Cd exposure differentially altered genes important in the regulation of the ECM favoring cancer, migration and invasion. With the advent of live high-content imaging, systematic morphodynamic assessments have great potential to serve as sensitive and feasible endpoints to evaluate the adverse cellular impacts of toxicants of environmental concerns and to visualize the biological behavior associated with cancer progression. One limitation of this study was that it was constrained by the amount of manual work required for image processing of the imaging results; therefore, the findings of the morphodynamic studies were based on a single cell culture. We are hoping that with the evolution of the Imaris software, the labor-intensive image processing steps will no longer be necessary, and we will be able to replicate the findings using multiple cell cultures cost effectively and involving less manual labor in the future. Encouragingly, our mRNA profiling analysis was based on three independently treated and untreated cultures. We observed minimal variation of mRNA expression in the three independent cultures indicating that each culture was representative. In our previous dose-response studies, with uterine fibroid cells being exposed to CdCl_2_ at concentrations of 10^−4^ µM to 200 µM, significant proliferative effects were observed at doses of 0.1 µM to 20 µM at 24 h, 48 h and 72 h [50]. In human tissues, the concentration of Cd is dependent on the age, sex, occupation, exposure duration, and smoking status of the individual and has been reported to range from approximately 0.9 µM in muscle up to 356 µM in the kidney cortex [51,52,53]. Therefore, due to Cd’s bioacculumative properties and its long half-life, concentrations of Cd present in human tissues and organs could be much higher than 10 µM.

## 5. Conclusions

We have demonstrated that prolonged Cd exposure not only stimulated cellular migration but also significantly enhanced heterogeneity of several cellular morphodynamic features such as nuclear size, shape, and speed using a time-series analysis. Long-term Cd exposure also altered cellular migration patterns such as migration directionality (track straightness) and nuclear orientation relative to track direction. The above Cd-induced alterations in benign uterine fibroid cells may be prerequisites or key intermediate steps necessary for cancer progression induced by heavy metals.

With the advent of live high-content imaging, we have developed a prototype to quantitatively analyze morphodynamic changes associated with chemical exposures using confocal microscopy, the Imaris 4D Image analysis package and R for assessing large datasets (more than 32,000 nuclear objects each). Further optimization of the morphodynamic analysis pipeline, especially the accuracy for object segmentation, will have great potential in expanding the use of this technology as a sensitive, feasible, and cost-effective means to facilitate systematic toxicological research and morphodynamics using in vitro cell culture models.

## Figures and Tables

**Table 1:** The Expressions of the mostly altered genes implicated in migration and invasion.

Gene Symbol	Gene Name	FDR (q-Value)	Fold Change	Location	Type(s)
*SPP1*	secreted phosphoprotein 1	1.11 × 10^−3^	15.55	Extracellular Space	cytokine
*MMP3*	matrix metallopeptidase 3	1.39 × 10^−5^	16.76	Extracellular Space	peptidase
*SULF1*	sulfatase 1	3.63 × 10^−7^	−16.67	Cytoplasm	enzyme

Note: This table was derived from IPA analysis of the NanoString data. The sample cells had been treated with Cd for 6 months. False discovery rate (FDR).

**Table 2:** Differential Expression of ECM and MMP transcripts between CR-LM6 and ht-UtLM6.

Symbol	Entrez Gene Name	FDR (q-Value)	Fold Change	Location
*MMP1*	matrix metallopeptidase 1	1.85 × 10^−4^	7.93	Extracellular Space
*MMP3*	matrix metallopeptidase 3	1.39 × 10^−5^	16.76	Extracellular Space
*MMP10*	matrix metallopeptidase 10	2.15 × 10^−2^	2.91	Extracellular Space
*COL1A2*	collagen type I alpha 2 chain	1.48 × 10^−2^	−3.03	Extracellular Space
*COL3A1*	collagen type III alpha 1 chain	1.10 × 10^−3^	−4.49	Extracellular Space
*COL4A2*	collagen type IV alpha 2 chain	5.34 × 10^−3^	−2.49	Extracellular Space
*COL5A2*	collagen type V alpha 2 chain	2.99 × 10^−2^	−2.57	Extracellular Space
*COL6A1*	collagen type VI alpha 1 chain	1.71 × 10^−2^	−2.01	Extracellular Space
*COL7A1*	collagen type VII alpha 1 chain	7.97 × 10^−3^	−3.33	Extracellular Space
*COL18A1*	collagen type XVIII alpha 1 chain	1.10 × 10^−2^	−2.04	Extracellular Space
*LAMA3*	laminin subunit alpha 3	2.82 × 10^−2^	−2.38	Extracellular Space
*LUM*	lumican	7.15 × 10^−3^	−2.36	Extracellular Space

Note: This table was derived from IPA analysis of the NanoString data. The sample cells had been treated with Cd for 6 months.

## Data Availability

The data from the NanoString RNA profiling experiments are publicly available at NCBI Gene Expression Omnibus under accession number GSE178790.

## Supplementary Materials

The following supporting information can be downloaded at: https://www.mdpi.com/article/10.3390/biomedicines10040917/s1, Supplementary Video S1: Nuclear migration track and nuclear orientation of Ht-UtLM6 (Track ID 32696). Supplementary Video S2: Nuclear migration track and nuclear orientation of CR-LM6 (Track ID 33806).
